# Supplementary material for: MetaRibo-Seq measures translation in microbiomes
Source: Nat Commun. 2020 Jun 29;11:3268. doi: 10.1038/s41467-020-17081-z (PMC7324362; doi:10.1038/s41467-020-17081-z)
Supplement: Supplementary file 10 — Supplementary Data 7 [file 41467_2020_17081_MOESM10_ESM.zip › File2/Confidence_High_Taxonomy/96639_out.krona.html]

Javascript must be enabled to view this page.

members
magnitude
magnitudeUnassigned
count
unassigned
taxon
rank

96639\_out

2

2
superkingdom
2

976
2
phylum

117743
class
1

order
1
200644

family
1
49546

1016
1
genus

1227265

SRS075771\_contig\_number\_contig-100\_21163.21164
species
1

200643
1
class

1
order
171549

family
1
171551

1
genus
836


SRS148520\_contig\_number\_contig-100\_22695.22696
997829
1
species
